# Effects of cancer-induced cachexia and administration of l-glutathione on the intestinal mucosa in rat

**DOI:** 10.1007/s00726-024-03391-9

**Published:** 2024-04-12

**Authors:** Sabrina Silva Sestak, Fabiana Galvão da Motta Lima, Ana Paula de Oliveira, Letícia Ganem Rillo Paz Barateiro, Flávia Cristina Vieira-Frez, Sara Raquel Garcia de Souza, Flávia Alessandra Guarnier, Juliana Vanessa Colombo Martins Perles, Jacqueline Nelisis Zanoni

**Affiliations:** 1 Department of Physiology, Laboratory of Enteric Neural Plasticity, State University of Maringá, O33 Block, Colombo Avenue, 5790, Maringá, Paraná CEP 87020-900 Brazil; 2 Department of Morphological Sciences, State University of Maringá, Maringá, Paraná Brazil; 3 Department of Pathology Sciences, State University of Londrina, Londrina, Paraná Brazil

**Keywords:** Antioxidant, Duodenum, Oxidative stress, Walker-256 tumor

## Abstract

Walker-256 tumor is an experimental model known to promote cachexia syndrome, oxidative stress, and systemic inflammation. This study evaluated the duodenal mucosa of rats with Walker-256 tumor administered with 1% l-glutathione, intending to evaluate the damage caused by cancer-associated cachexia in the gastrointestinal tract and the effects of antioxidant administration on mucosal protection. Twenty-four 55-day-old male Wistar rats were distributed into four groups: control (C); control administered with 1% l-glutathione (C-GSH); Walker-256 tumor (W) and Walker-256 tumor administered with 1% l-glutathione (W-GSH). After 14 days of treatment, the duodenum was harvested for morphometric analysis of the mucosa, proliferation, apoptosis, immunostaining of varicosities immunoreactive (IR) to vasoactive intestinal peptide (VIP) and 5-HT-IR cells, and quantification of mast cells and goblet cells. Walker-256 tumor-bearing rats showed cachexia syndrome, mucosal atrophy, reduced cell proliferation, reduced 5-HT-IR cells, and increased goblet cells and VIPergic varicosities, which were not reversed by l-glutathione. On the other hand, l-glutathione caused a reduction of cells in apoptosis and mast cell recruitment, demonstrating a partial recovery of the damage detected in the intestinal mucosa.

## Introduction

Walker-256 carcinosarcoma is an animal tumor model that induces several metabolic changes leading to systemic inflammation and cachexia syndrome (Angelo and Oliveira 2009). Cachexia involves the involuntary loss of weight and muscle mass, changes in taste sensitivity, early satiety, weakness, and atrophy of visceral organs and adipose tissue (Teixeira 2002; Petruzzelli et al. 2014). Cachexia is strongly related to systemic inflammation, as inflammatory cytokines induce muscle wasting even in the presence of adequate nutrition (Onesti and Guttridge 2014).

Cancer also promotes the formation of reactive oxygen species leading to oxidative stress (Sosa et al. 2013; Schumacker 2015). Reactive oxygen species can cause cellular damage in the gastrointestinal tract and affect the intestinal barrier, increasing permeability (Basilicata et al. 2018).

To control the levels of reactive oxygen species, the body relies on antioxidants that can delay or inhibit the oxidation of oxidizable substrates, thereby avoiding and/or mitigating the harmful effects of reactive species (Vasconcelos et al. 2015). One example is reduced glutathione (GSH). GSH is a tripeptide (γ-l-glutamyl-l-cysteinyl-glycine) that acts directly or indirectly in many biological processes. In the intestine, GSH participates in the elimination of lipid peroxides from food, which can compromise mucosal metabolic pathways, cause enterocyte dysfunction, and contribute to developing intestinal pathologies such as inflammation and cancer (Aw 2005).

Thus, this study aimed to evaluate the effects of 1% l-glutathione administration on the intestinal mucosa given the implications generated by the Walker-256 tumor.

## Materials and methods

### Ethics, experimental groups, and cancer induction

Twenty-four 55-day-old male Wistar rats (*Rattus norvegicus*) from the Central Animal Facility of the State University of Maringa were used in this work. The animals were randomly distributed into four groups (*n* = 6): control (C), control administered with 1% l-glutathione (C-GSH), Walker-256 tumor (W), and Walker-256 tumor administered with 1% l-glutathione (W-GSH). During the treatment, the animals were kept in polypropylene boxes measuring 40 × 33 × 17 cm (length, width, and height) and under controlled environmental conditions (temperature of 22º ± 2 ºC and illumination cycle 12 light/12 dark), receiving feed and water ad libitum.

The animals in the C-GSH and W-GSH groups received l-glutathione (Active Pharmaceutica, SC, Brazil) introduced in standard feed at a concentration of 1% (1 g/100 g of feed), while animals in the C and W groups received standard rodent chow (Nuvilab®, Colombo, PR, Brazil). l-Glutathione was incorporated into standard ground feed and later remodeled into pellets which remained in an oven at 55 °C for 72 h for drying (Hermes-Uliana et al. 2014). Food consumption was analyzed daily and the animals were weighed on the first and last day of the 14-day experimental period.

Cancer induction (W and W-GSH groups) was performed according to the method established by Guarnier et al. (2010). A suspension of 8.0 × 10^7^ viable tumor cells in 0.5 ml of 16.5 mM phosphate-buffered saline (PBS), pH 7.3, was subcutaneously injected into the right flank of each animal. For the control groups, the same volume of PBS (16.5 mM, pH 7.4) was applied in the same anatomical site of each animal.

## Collection and processing of samples

Before euthanasia, the animals were maintained in a 12 h fasting period. Then, the animals received an intravenous injection of vincristine sulfate (0.5 mg/kg body weight––Tecnocris®, Zodiac Laboratories, SP, Brazil) 2 h before euthanasia. Vincristine was used to study the metaphasic index and the best evidence of VIPergic nerve fibers in the intestinal mucosa. Sample collection time occurred around 1 o'clock in the afternoon, respecting the circadian cycle of cell proliferation. After the vincristine period, the animals were euthanized under intraperitoneal anesthesia with lidocaine (10 mg/kg body weight––Lidovet, Laboratório Bravet LTDA, RJ, Brazil), followed by the general anesthetic thiopental (150 mg/kg body weight––Abbot Laboratories, Chicago, IL, USA). After a celiotomy, the duodenum was removed and cut into two 2 cm segments which were used for histological and immunohistochemical techniques. The duodenum for both techniques was opened along the mesenteric border, adhered to a styrofoam plate with the mucosal surface facing upwards, and carefully washed with PBS (0.1 M, pH 7.4).

## Calculation of cachexia index

The tumor from the W and W-GSH groups was removed and weighed to calculate the cachexia index. The index was obtained through Eq. (1), in which only animals that presented a loss of body mass greater than 10% were considered cachectic (Guarnier et al. 2010).

1 $$\% {\text{ loss of body mass }} = \, \left( {{\text{ibm}}{-}{\text{fbm }} + {\text{ tm }} + {\text{ mgc}}} \right){ 1}00 \, / \, \left( {{\text{ibm }} + {\text{ mgc}}} \right),$$

where ibm is the initial body mass of the animal, fbm is the final body mass, tm is the tumor mass and mgc is the mean mass gain of the control group (Martins et al. 2016).

## Sample processing for histological techniques

The duodenum was fixed in Bouin (picric acid, formaldehyde, and acetic acid) for 6 h at room temperature (RT). The samples were then dehydrated with increasing series of alcohols and embedded in paraffin to obtain 4-μm-thick semi-serial sections, using a microtome Leica RM2125 (Leica Biosystems, Nussloch, Germany).

For coloring the samples, the tissue underwent deparaffinization steps in xylol and hydration with decreasing series of alcohols. Then the cuts were stained with hematoxylin and eosin (HE), periodic acid–Schiff (PAS), Blue Toluidine, and Fuchsin Orange G, with specific times and washes for each type of dye. Next, the cuts were dehydrated in alcohol, diaphanized, and mounted in slides with permount. HE staining was used for the analysis of morphometry of the intestinal mucosa and cell proliferation; PAS was used for evidencing neutral mucin-producing goblet cells and the Toluidine Blue and Fuchsin Orange G for evidencing mast cells.

## Samples processing for immunohistochemical techniques

The duodenum was fixed in Zamboni (picric acid and buffered paraformaldehyde) for 18 h at 4 °C, then washed in PBS (0.1 M, pH 7.4) for 12 h, changing the PBS in the sixth hour. The material underwent a cryoprotection process solution in PBS (0.1 M, pH 7.4) and 18% sucrose (Casa da Química, São Paulo, Brazil) for 24 h at 4 ºC. Afterward, the samples were included in a solution of optimum cutting temperature (O.C.T––Fisher HealthCare, Tissue Plus®, USA) and underwent instantaneous freezing in liquid nitrogen (− 196 °C), being stored at − 80 °C. The samples were sectioned using the Leica CM1850 cryostat (Leica Biosystems, Nussloch, Germany), in 10 μm semi-serial sections and adhered to slides prepared with poly-l-lysine solution (Sigma–Aldrich Co., Missouri, USA). These cuts were intended for immunohistochemical techniques to evidence immunoreactive (IR) varicosities to vasoactive intestinal peptide (VIP) and 5-HT-IR and Caspase-3-IR cells.

## Immunohistochemistry for evidencing VIP-IR varicosities and double immunostaining of 5-HT-IR and caspase-3-IR cells

Histological sections were washed with PBS (0.1 M, pH 7.4) + Triton X-100 0.5% (Sigma–Aldrich Co., Missouri, USA)–for 10 min. Followed by incubation in blocking solution (PBS; 0.1 M, pH 7.4 + 0.5% Triton X-100 + 2% bovine serum albumin (BSA-Sigma–Aldrich Co., Missouri, USA) + 10% donkey serum) for 1 h RT. With subsequent incubation in VIP or 5-HT + Caspase-3 (Table 1) primary antibody in PBS^+^ solution (PBS; 0.1 M, pH 7.4 + Triton X-100 0.5% + 2% BSA + 2% donkey or goat serum) at 4 ºC, being donkey serum used for immunohistochemistry for VIP and goat serum for 5-HT + Caspase-3. After incubation, three washes with PBS (0.1 M, pH 7.4) + 0.5% Triton X-100 were performed for 5 min each. Then the samples were incubated with the secondary antibody (Table 1), remaining at RT also in a PBS + solution. At the end of incubation, three new washes with PBS (0.1 M, pH 7.4) were performed for 5 min each with subsequent slide mounting in 10% buffered glycerol. The methodology was performed in a humid chamber.

**Table 1:** Specifications of primary and secondary antibodies used for immunohistochemical techniques

Staining	Primary antibody	Origin	Dilution	Incubation time
VIP-IR varicosities	Rabbit anti-VIP; S0390	Sigma, USA	1:200	24 h
5-HT-IR cells	Rabbit anti-5-HT; S5545	Sigma, USA	1:400	72 h
Caspase-3-IR cells	Goat anti-Caspase-3; Sc-1226	Santa Cruz, USA	1:150	72 h
	**Secondary antibody**			
VIP-IR varicosities	Alexa Fluor 488 (Donkey anti-rabbit); A-21206	Molecular Probes, Invitrogen, USA	1:500	2 h
5-HT-IR cells	Alexa Fluor 488 (Donkey anti-rabbit); A-21206	Molecular Probes, Invitrogen, USA	1:500	4 h
Caspase-3-IR cells	Alexa Fluor 568 (Donkey anti-goat); A-11057	Molecular Probes, Invitrogen, USA	1:500	4 h

## Image acquisitions

The images for analysis of goblet cells, mast cells, and the morphometry of the mucosa were captured using a high-resolution camera––Moticam® 2500 5.0 MegaPixel (Motic China Group Co. Ltd, Xiamen, China) coupled with an optical microscope––Motic® BA400 (Motic China Group Co. Ltd, Shanghai, China) and transferred to a computer through the software––Motic Images Plus® 2.0 ML (Motic China Group Co., Xiamen, China). For immunohistochemistry, the images were captured using a high-resolution camera––MoticamPro® 252B (Motic China Group Co. Ltd, Xiamen, China) coupled to a fluorescence optical microscope––Olympus® BX40 (Olympus Co., Japan), transferred to a computer through the software––Motic Images Plus® 2.0ML (Motic China Group Co. Ltd, Xiamen, China). The analyses were performed using Image-Pro Plus version 4.5.0.29 analysis software (Media Cybernetics, Silver Spring, MD, USA) and SoftGoitaca 3.43 *software*.

## Intestinal mucosa morphometry and metaphasic index

We selected well-oriented longitudinal sections to perform the intestinal mucosa morphometry analysis. Thirty villi, thirty crypts and thirty measurements of height were used to determine depth and height in images randomly captured with a 10 × objective. The crypt depth comprises the extent of the crypt-villus junction to the base of the crypt. The height of the villi extends from the crypt–villi junction to the apex of the villi. While the height of the mucosa varies from muscular mucosa to the top of the villi. Adapted from Martins et al. (2016).

The metaphasic index was calculated using an optical microscope—Motic® BA400 (Motic China Group Co. Ltd, Shanghai, China), 100 × objective. The percentage of metaphasic cells was obtained by calculating the number of counted metaphasic nuclei × 100/total number of counted nuclei per animal. The total number of counted nuclei was 2500 per animal. Adapted from Martins et al. (2016).

## PAS + goblet cell and mast cell quantification

The quantification of PAS + goblet cells was performed on the epithelium of 30 villi and 30 crypts per animal (180 crypt/villus units per group). Otherwise, mast cell quantification was performed in the lamina propria of 30 villi per animal (180 villi per group) in images captured with a 20 × objective. Adapted from Martins-Perles et al. (2019).

## Morphometry and quantification of VIP-IR varicosities

The area (μm^2^) of 300 varicosities per animal was measured using images captured with a 40 × objective. The quantitative analysis was performed using stereology (fraction of points) using the SoftGoitaca 3.43 software (Sales et al. 2021). A total of 30 fields were randomly captured with a 40 × objective on the villi lamina propria. In the software, a mesh of 6510 points (distance of 11 pixels) was overlapped on the image, followed by automatic quantification of the number of points on the varicosities. The results were expressed as the percentage of incident points on the image, which can be understood as the percentage of varicosities present in the field.

## Quantification of 5-HT-IR, Caspase-3-IR, and 5-HT + Caspase-3-IR cells

The quantification of 5-HT-IR, Caspase-3-IR, and 5-HT + Caspase-3-IR cells was performed in 30 crypt epithelium, and the epithelium and lamina propria of 30 villi per animal, using the same fields. Based on Martins-Perles et al. (2019). Images were obtained using a 10 × objective.

## Correction factor for quantitative analysis for villi end crypt

We calculated a correction factor (CF) to match the heights of villi and depths of crypts from groups C-GSH, W, and W-GSH to group C, through Eqs. (2,3).

2 $${\text{NC}}_{{\text{x}}} { = }\frac{{{\text{M}}_{{\text{C}}} {\text{NC}}_{{\text{e}}} }}{{{\text{M}}_{{\text{e}}} }},$$

3 $${\text{CF = }}\frac{{{\text{NC}}_{{\text{x}}} }}{{{\text{NC}}_{{\text{e}}} }},$$

where NCx is the proportional number of cells to the corrected measure (villi or crypt) of the different groups (C-GSH, W, W-GSH) compared with the control, Mc is the measure (villus or crypt) of the control group, NCe is the number of cells studied in the different groups (C-GSH, W, W-GSH) and Me is the measure (villus or crypt) of the different groups studied (C-GSH, W, W-GSH). This CF was applied in C-GSH, W, and W-GSH by multiplication.

## Statistical analysis

Results were expressed as the mean ± standard error of the mean (SEM). For the morphometric and quantitative analysis, the data comparison was obtained by variance analysis (block design) followed by Fisher's test. For physiological analyses, the data comparison was obtained by One-Way (ANOVA) followed by Fisher's test. All the analyses were performed at Statistica 7.0 (StatSoft) software. The significance level adopted was *p* ≤ 0.05.

## Results

### Pathophysiologic parameters

The W group showed a 24% reduction in daily consumption of food and a simultaneous reduction of 73% in mass gain and 15% in body mass (*p* < 0.05). The W and W-GSH groups showed similarities in mass gain and final body mass, although daily consumption was significantly lower in the W-GSH group when compared to W (Table 2). Similar results between W and W-GSH groups were also observed in tumor development. In both groups, Walker-256 cells promoted solid tumor formation in the right flank and also induced cachexia syndrome. The cachexia index, as well as the tumor weight in the W-GSH group, did not present a significant difference from the W group (Table 2). All physiological parameters analyzed were similar between the C and C-GSH groups (*p* > 0.05, Table 2).

**Table 2:** Physiological parameters of the experimental groups: control (C); control administered with 1% l-glutathione (C-GSH); Walker-256 tumor (W); Walker-256 tumor administered with 1% l-glutathione (W-GSH)

Parameters	C	C-GSH	W	W-GSH
Initial body mass (g)	208.8 ± 3.2	219.0 ± 15.7	219.5 ± 3.4	203.2 ± 8.8
Final body mass (g)	283.0 ± 5.4	288.8 ± 18.3	239.5 ± 6.9^*^	223.3 ± 7.5
Mass gain (g)	74.2 ± 5.2	69.8 ± 16.3	20.0 ± 6.3^**^	20.2 ± 7.1
Food intake (g/animal/day)	26.7 ± 0.7	25.8 ± 0.8	20.3 ± 0.6^***^	17.8 ± 0.9^###^
Tumor mass (g)	–	–	27.0 ± 1.1	29.4 ± 2.9
Cachexia index (%)	–	–	27.7 ± 2.4	29.1 ± 2.7

Values expressed as mean ± SEM. * *p* < 0.05, ** *p* < 0.001, *** *p* < 0.0001 when compared with group C; ### *p* < 0.0001 when compared with the W group. *n* = 6 animals per group

## Intestinal mucosal morphometry and metaphasic index

A reduction in mucosal height and crypt depth was observed in the W group (vs. C, *p* < 0.05, Table 3). These parameters were even lower in the W-GSH group (vs. W, *p* < 0.02, Table 3). All morphometric measurements of the duodenal wall were lower in the C-GSH group when compared to the C group (*p* < 0.05, Table 3).

**Table 3:** Duodenal mucosa morphometry of the experimental groups: control (C); control administered with 1% l-glutathione (C-GSH); Walker-256 tumor (W); Walker-256 tumor administered with 1% l-glutathione (W-GSH)

Parameters	C	C-GSH	W	W-GSH
Mucosa height (µm)	517.9 ± 10.3	428.8 ± 7.9^***^	493.1 ± 7.2^*^	433.0 ± 9.1^###^
Villus height (µm)	284.9 ± 7.2	250.9 ± 5.9^***^	272.9 ± 5.0	260.7 ± 5.9
Crypt depth (µm)	164.1 ± 4.4	126.4 ± 3.4^***^	142.9 ± 3.8^**^	130.5 ± 4.2^#^

Values expressed as mean ± SEM. * *p* < 0.05, ** *p* < 0.001, *** *p* < 0.0001 when compared with group C; # *p* < 0.05, ## *p* < 0.001, ### *p* < 0.0001 when compared with the W group. *n* = 6 animals per group

**Fig. 1:**
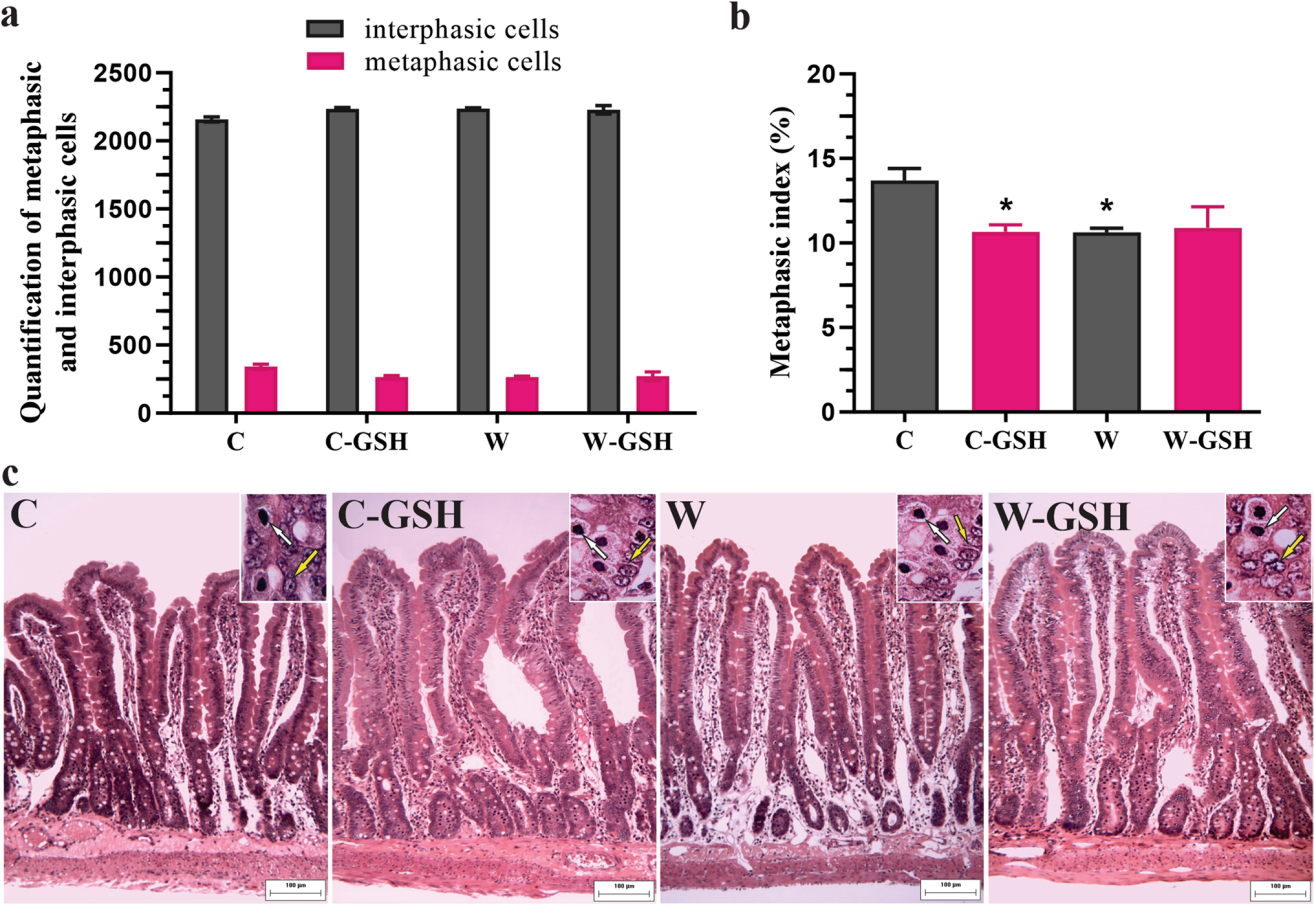
Metaphasic index. **a** Quantification of metaphasic and interphasic cells, **b** Metaphasic index (%), **c** Photomicrograph of duodenal mucosa stained with hematoxylin and eosin. The white arrow indicates metaphasic cell and the yellow arrow indicates interphasic cell in the crypt region. Control (C); control administered with 1% l-glutathione (C-GSH); Walker-256 tumor (W); Walker-256 tumor administered with 1% l-glutathione (W-GSH). The results were expressed as mean ± SEM. *p < 0.05 when compared to group C.* n* = 6 animals per group. Scale bar 100 μm

The metaphasic index was obtained with the ratio of interphasic and metaphasic cells (Fig. 1a). W and C-GSH groups presented the same reduction in the metaphasic index (22% vs. C, *p* < 0.01; Fig. 1b). l-glutathione administration did not alter the cell proliferation in the W-GSH group (Fig. 1b). Photomicrographs of duodenal mucosa are demonstrated in Fig. 1c.

## Number of PAS + goblet cells and mast cells in the mucosa

**Fig. 2:**
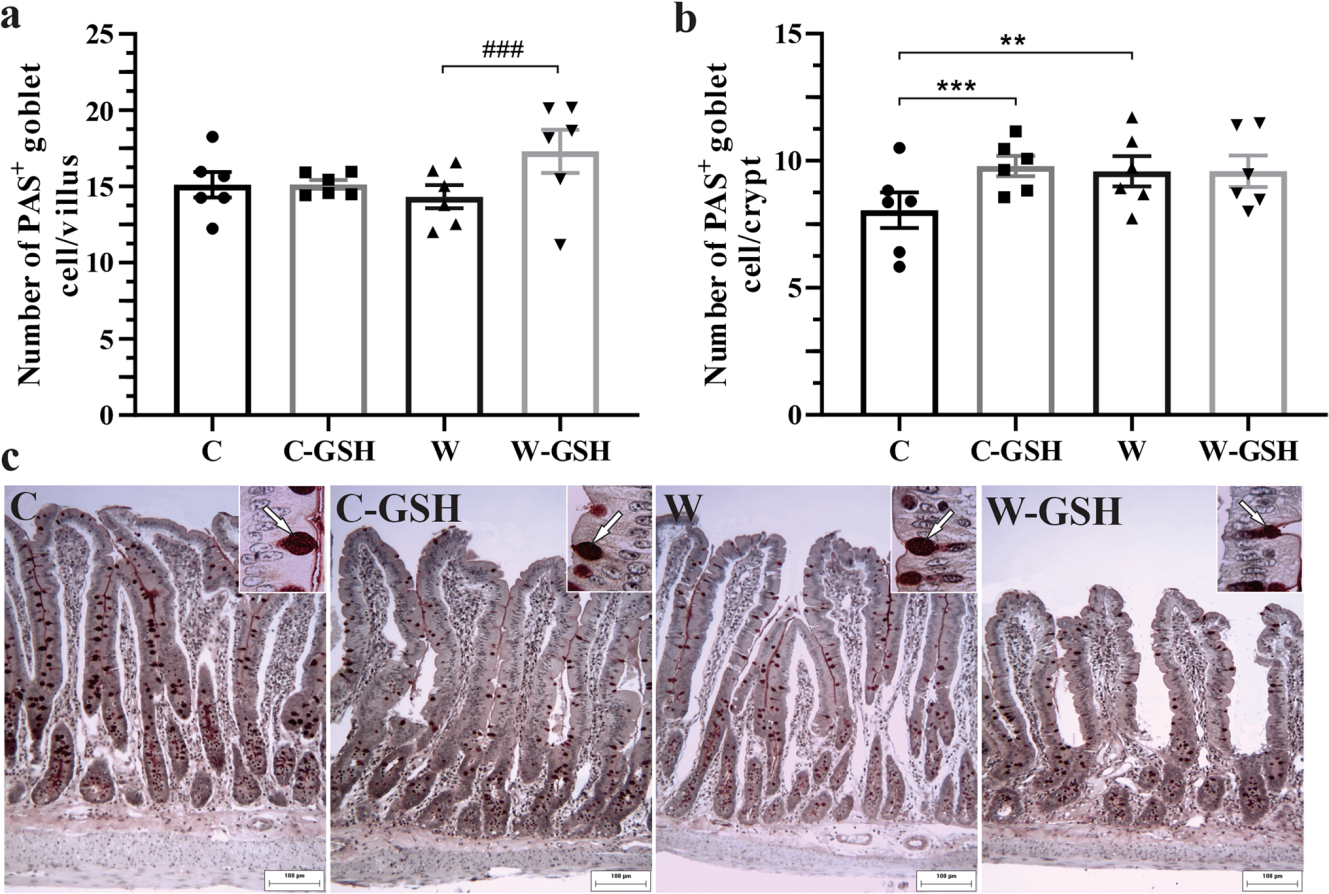
Number of PAS^+^ goblet cells. **a** Number of PAS^+^ goblet cell/villus, **b** Number of PAS^+^ goblet cell/crypt, **c** Photomicrograph of duodenal mucosa stained with periodic acid–Schiff. The white arrow indicates PAS^+^ goblet cell in the epithelium. Control (C); control administered with 1% l-glutathione (C-GSH); Walker-256 tumor (W); Walker-256 tumor administered with 1% l-glutathione (W-GSH). The results were expressed as mean ± SEM. ** *p* < 0.001, *** *p* < 0.0001 when compared to group C; ### *p* < 0.0001 when compared to the W group* n* = 6 animals per group. Scale bar 100 μm

In the villi, the number of PAS + goblet cells was similar the C, C-GSH and W groups, meanwhile, it was higher in W-GSH, with a 21% increase compared to in W (*p* < 0.0001; Fig. 2a). In the crypts, it was observed a 19% increase in the number of PAS + goblet cells at W (vs. C; *p* < 0.0005). Administration with l-glutathione did not alter this (W-GSH vs. W, Fig. 2a), on the other hand, in healthy animals, there was a 21% increase in the number of PAS + goblet cells (vs. C; *p* < 0.0005; Fig. 2b). Photomicrographs of duodenal mucosa showing PAS + goblet cells are represented in Fig. 2c.

**Fig. 3:**
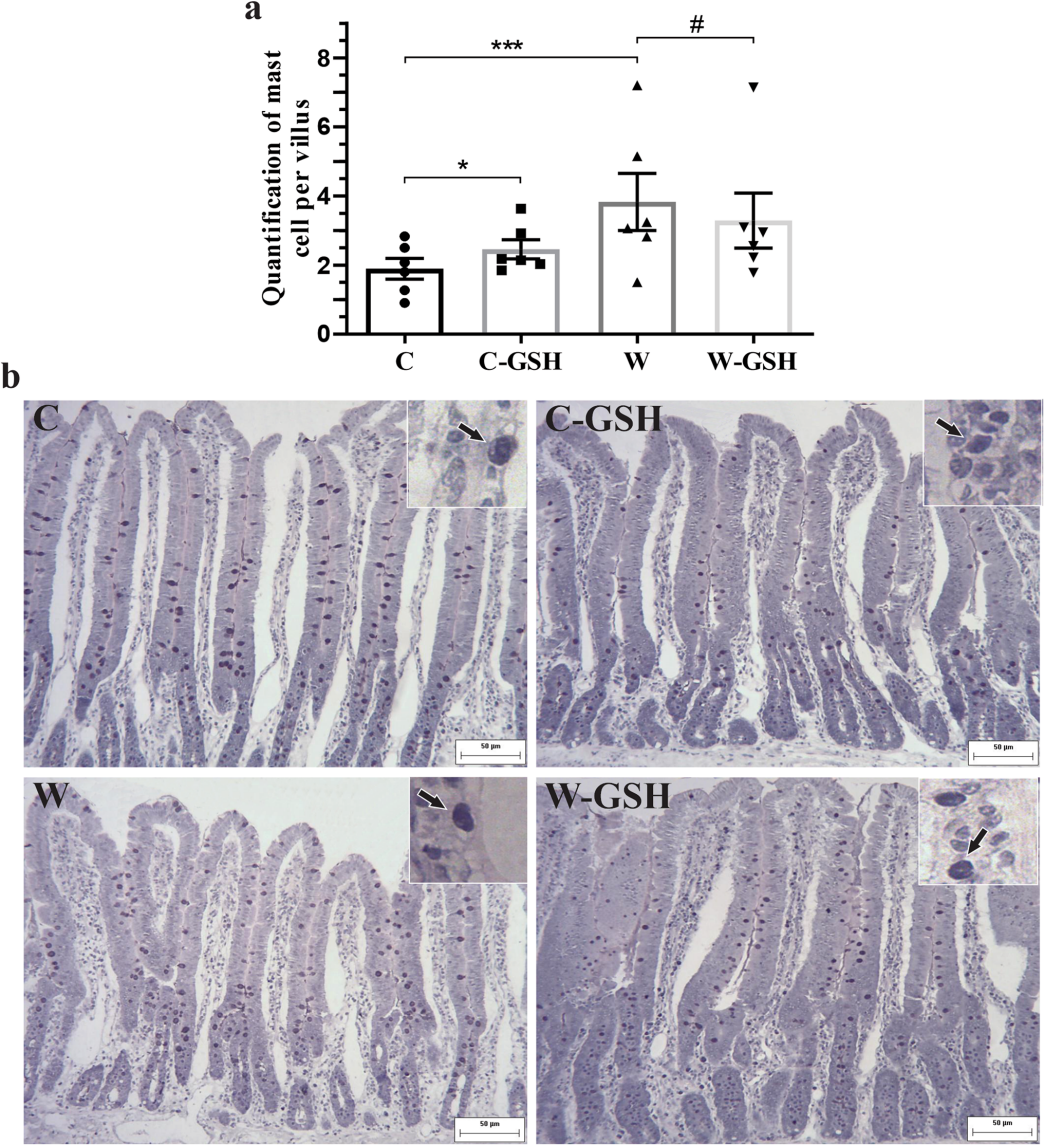
Quantification of mast cells. **a** Quantification of mast cells per villus, **b** Photomicrograph of duodenal mucosa stained with Toluidine Blue and Fuchsin Orange G. White arrow indicates mast cell in the lamina propria. Control (C); control administered with 1% l-glutathione (C-GSH); Walker-256 tumor (W); tumor of Walker-256 administered with 1% l-glutathione (W-GSH). The results were expressed as mean ± SEM. * *p* < 0.05, ** *p* < 0.0001 when compared with group C; # *p* ≤ 0.05 when compared to the W group.* n* = 6 animals per group. Scale bar 50 μm

We observed a 30% increase in the number of mast cells in the C-GSH group and 102% in the W group (vs. C; *p* < 0.0001; Fig. 3a). In W rates, the administration of l-glutathione caused a 14% reduction in the number of mast cells (*p* = 0.05; Fig. 3b). Photomicrographs of duodenal mucosa showing mast cells are represented in Fig. 3b.

## VIP-IR varicosities in the duodenal mucosa

**Fig. 4:**
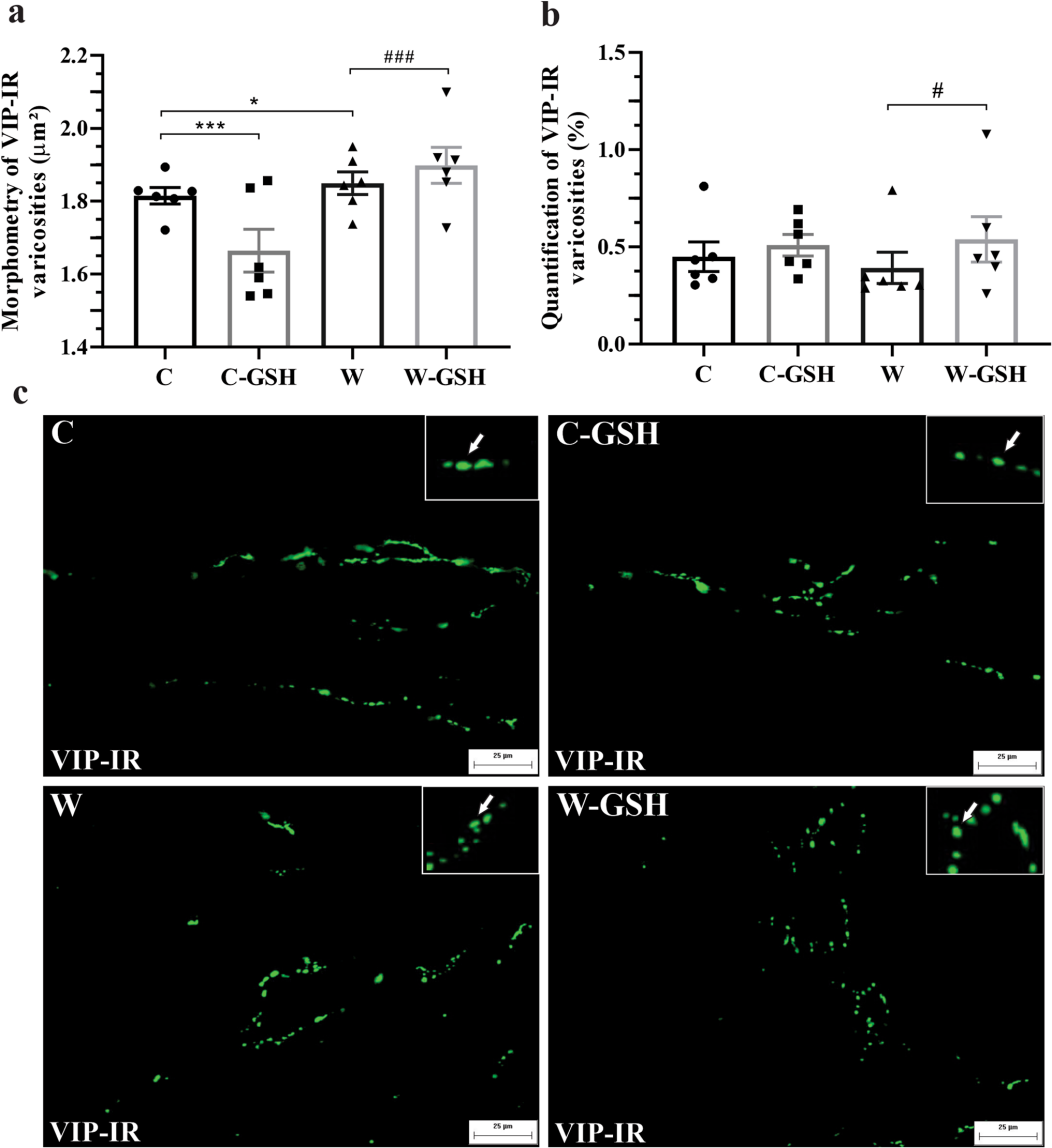
Morphometry and quantification of VIP-IR varicosities. **a** Morphometry of VIP-IR varicosities (μm^2^), **b** Quantification of VIP-IR varicosities (%), **c** Photomicrograph of VIP-IR varicosities present in the duodenal mucosa. The white arrow indicates VIP-IR varicosity. Control (C); control administered with 1% l-glutathione (C-GSH); Walker-256 tumor (W); Walker-256 tumor administered with 1% l-glutathione (W-GSH). The results were expressed as mean ± SEM. * *p* < 0.05, *** *p* < 0.0001 when compared with group C; # *p* < 0.05, ### *p* < 0.0001 when compared with the W group.* n* = 6 animals per group. Scale bar 25 μm

In the control rats, the area of VIP-IR varicosities was significantly reduced in presence of GSH (vs. C; *p* < 0.0001; Fig. 4a). In the W group, we observed an increase in the areas of VIP-IR varicosities (W vs. C; *p* = 0.02; Fig. 4a). In contrast, the quantification showed no significant difference in the C-GSH and W groups (vs. C; Fig. 4b).

Administration of glutathione in cachectic animals caused overexpression of VIP-IR varicosities compared to the group without treatment since the area and quantity of these varicosities were significantly higher (W-GSH vs. W; *p* < 0.001; Fig. 4a, b).

## Quantification of 5-HT-IR, Caspase-3-IR, and 5-HT + Caspase-3-IR cells

**Fig. 5:**
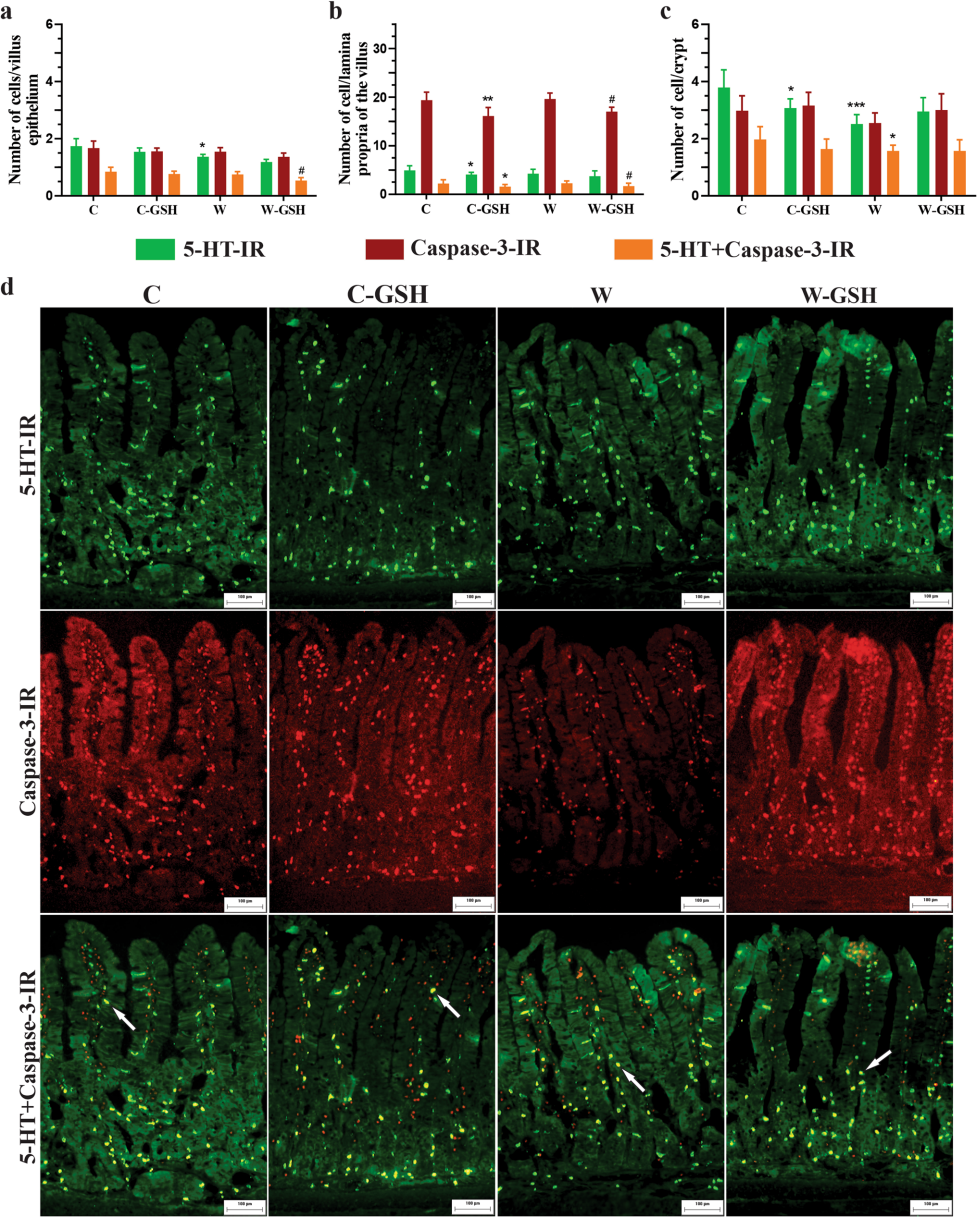
Quantification of 5-HT-IR, Caspase-3-IR, and 5-HT + Caspase-3-IR cells at epithelium and lamina propria of the villus and in the crypt. **a** Number of cells/villus epithelium, **b** Number of cell/lamina propria of the villus, **c** Number of cell/crypt, **d** Photomicrograph of the duodenum mucosa showing 5-HT-IR, Caspase-3-IR, and 5-HT + Caspase-3-IR cells. The white arrow indicates 5-HT + Caspase-3-IR cell. Control (C); control administered with 1% l-glutathione (C-GSH); Walker-256 tumor (W); Walker-256 tumor administered with 1% l-glutathione (W-GSH). Results were expressed as mean ± SEM. * *p* < 0.05, ** *p* < 0.001, *** *p* < 0.0001 when compared with group C; # *p* < 0.05 when compared with the W group.* n* = 6 animals per group. Scale bar 100 μm

Our study showed that in the C-GSH group, there was no change in the number of 5-HT-IR cells in the villi epithelium (Fig. 5a), although we observed a reduction in the number of 5-HT-IR cells in the crypt region and the lamina propria (C-GSH vs. C; *p* < 0.043; Fig. 5b, c). The W group showed no difference in the number of 5-HT-IR cells in the lamina propria when compared to the C group. However, it exhibited significant reductions of 25% and 34% in the epithelium and crypts, respectively (Fig. 5a, c). The W-GSH group showed similarity in the number of 5-HT-IR cells in all analyzed regions (W-GSH vs. W).

Regarding the number of Caspase-3-IR cells, the C-GSH group displayed a reduction only in lamina propria (vs. C; *p* < 0.0002). The W group exhibited similarity in the number of Caspase-3-IR cells in the three regions analyzed when compared to the control (C). However, administration of l-glutathione to cancer animals reduced apoptosis by 13% in the lamina propria (W-GSH vs. W; *p* = 0.003; Fig. 5b).

In the W group, there was a significant reduction of 20% of 5-HT + Caspase-3-IR cells only in the crypts (Fig. 5c). l-glutathione reduced the number of 5-HT + Caspase-3-IR cells by 28% in the lamina propria (C-GSH vs. C; *p* < 0.01). Animals with cancer presented reductions of 29% and 26% of cells in the villi epithelium and lamina propria, respectively (W-GSH vs. W; *p* < 0.05; Fig. 5a, b).

## Discussion

In this study, we observed progressive tumor development in the right flank of the animals, accompanied by the development of a cachexia syndrome. Similar results have been reported previously in the same model (Deminice et al. 2016; Vicentini et al. 2016; Martins et al. 2016). A decrease in food intake and mass gain was still observed in animals with Walker-256 tumor, which was also found by Marega et al. (2018). These results were expected considering that cachectic animals tend to present malnutrition (Vaughan et al. 2013).

The untreated cachectic animals showed atrophy of the duodenal mucosa. Stem cells present in the crypts are essential for self-renewal of the epithelium (Delgado et al. 2016), where altered cell proliferation may have been reflected in reduced 5-HT-IR epithelial cells in the villus and crypt. Martins et al. (2016) also evidenced reduced cell proliferation and subsequent intestinal mucosal atrophy in the duodenum of animals bearing Walker-256 tumors. These changes are associated with reduced absorptive processes, contributing to a consequent reduction in mass gain and cachexia.

During cancer development, there is an association between increased inflammatory cytokines and the induction of reactive oxygen species formation, which characterizes a state of systemic oxidative stress (Sosa et al. 2013; Schumacker 2015). Oxidative stress in turn induces inhibition of cell proliferation by activating the nuclear translocation of NF-kB, which leads to the expression of IL-1β, which promotes inhibition of cell proliferation of epithelial cells in the duodenum (Gezginci‑Oktayoglu et al. 2010).

Although oxidative stress induces intestinal apoptosis (Ruan et al. 2018), our results were different. The maintenance of Caspase-3-IR cell numbers was demonstrated when evaluated alone or in double labeling with the 5-HT-IR cell in all regions analyzed, except for Caspase-3-IR + 5-HT-IR in the crypt, where the reduction was observed. The maintenance and/or reduction of apoptotic cells may be a compensatory mechanism for reduced cell proliferation, since the balance of epithelial renewal is essential for intestinal morphology and function (Williams et al. 2015) and, when compromised, affects the integrity of the intestinal barrier (Martini et al. 2017). In IEC-6 cells in vitro, low concentrations of 5-HT have been observed to inhibit apoptosis of these cells (Dong et al. 2016), and although the concentration of 5-HT was not evaluated in our study, we can infer that the reduction in cells number may be related to a lower concentration of 5-HT and consequently reduced apoptosis in W animals.

In untreated cachectic animals, we also observed an increase in the area of VIPergic varicosities, with no change in density. This may reflect changes in VIP-IR neurons in the submucosal plexus, as demonstrated in this model of cachexia (Vicentini et al. 2016). VIP negatively regulates some pro-inflammatory cytokines and can stimulate T-cell production and inhibit the pro-inflammatory effects of macrophages, thus contributing to the negative regulation of inflammation (Chandrasekharan et al. 2013).

In addition, VIP is strongly related to mast cells present in the mucosa, by being able to stimulate mast cell activation, to the point that mast cells can also release VIP (Sohn et al. 2013; Theoharides 2017). Studies mention that mast cells and VIP are closely related to the gastrointestinal barrier during inflammation (Sohn et al. 2013; Keita et al. 2013; Bednarska et al. 2017). This leads us to understand, as a disease defense mechanism/phenomenon, the expressive number of mast cells in cachectic animals.

The number of goblet cells is also influenced by VIPergic innervation (Sant’Ana et al. 2012; Wu et al. 2015; Schneider et al. 2018; Schwerdtfeger and Tobet 2020). This is consistent with our results, where the number of goblet cells in the intestinal epithelium of cachectic animals showed increased along with higher expression of VIPergic varicosities in the mucosa. The increase of PAS + cells in the mucosa of diseased animals was also demonstrated by Martins et al. (2016), and the authors suggest that such an increase is a compensatory mechanism to increase mucin production, strengthen the mucus layer, and provide greater protection for the intestine weakened by the systemic consequences of cancer.

GSH acts directly or indirectly in many biological processes, including protein synthesis, maintenance of intracellular redox balance and redox signaling, metabolism and protection cellular, detoxification, regulation of proliferation and apoptosis, and exerts immunobiological functions (Rover Júnior et al. 2001; Traverso et al. 2013; Lu 2013). Gender differences in GSH metabolism in aging and some pathologies, such as neurodegenerative diseases and diabetes, have already been described in the literature, with males being more susceptible to oxidative stress and decreased plasma GSH (Wang et al. 2003, 2020; Liu et al. 2005). The possible gender differences in GSH metabolism in cancer pathology are poorly understood, in this study, only male animals were adopted due to possible greater oxidative stress and GSH depletion, although future studies with female rats would be interesting to understand better the effects of GSH on the intestinal mucosa of female rats with cancer-induced cachexia.

The concentration of GSH in the intestine is due to intake (depending on the consumption of foods rich in cysteine, glycine, and glutamic acid) and bile output which is estimated to account for 50% of hepatic GSH (Circu and Aw 2012). Administration of 1% l-glutathione for 14 days increases GSH levels in the plasma and small intestine of animals with Walker-256 tumor (Oliveira et al. 2023), although it is not able to prevent the development of cachexia, as evidenced in this study, unlike its precursor l-glutamine that was used previously in the same experimental model (Vicentini et al. 2016; Martins et al. 2016).

l-glutathione also did not reverse the intestinal atrophy and inhibition of cell proliferation in animals in the W-GSH group. It was found that supplementation with 1% glutathione for 2 weeks restored intracellular GSH/GSSG balance without normalizing enterocyte proliferation in the duodenum of peroxide-lipid-induced mice (Tsunada et al. 2003). In contrast, in the same study, the authors showed that cell proliferation was restored after 4 weeks of glutathione supplementation. Thus, we believe that the experimental period of 14 days, adopted in this study, was too short a period to show changes in epithelial proliferation after administration of l-glutathione. It is important to highlight that the duration of the 14-day experiment adopted in this study is due to the aggressiveness of the model and the survival of the animals (Guaitani et al. 1982; Angelo and Oliveira 2009).

Even with intestinal atrophy, in W-GSH animals the number of 5-HT-IR cells was not altered, as well as the number of apoptotic cells in the epithelium of the villi and crypt. The reduction seen in the lamina propria of the villi indicates the antioxidant effect of l-glutathione since exogenous administration increases plasma and intestinal levels of GSH (Aw et al. 1991; Jordão Jr et al. 1998; Oliveira et al. 2023) assisting in the redox balance and avoiding oxidative stress that induces apoptosis, reflecting in the reduction of 5-HT + Caspase-3-IR cells in the epithelium and lamina propria of the villus.

The reduction in apoptosis of 5-HT-IR cells in the villus epithelium promoted by l-glutathione administration is a positive aspect because, according to Gershon (2013), enterochromaffin cells are classically known for their role as mechanoreceptors that secrete serotonin, which initiates peristaltic reflexes and is also essential for the gastrointestinal inflammatory response.

In the intestinal mucosa of W-GSH animals, an overexpression of VIP-IR varicosities was detected, and in a previous work of our group (Hermes-Uliana et al. 2014), an increased cell body of VIP-IR submucosal neurons was demonstrated in animals treated with l-glutathione in a model of diabetic neuropathy. It is worth noting that the nerve fibers present in the intestinal mucosa originate from neurons present in the plexuses responsible for controlling various epithelial functions (Fornai et al. 2018). The increased expression of VIP in the cachectic animals with and without treatment may be a response to the pathological state, as the intestinal mucosa is weakened. l-glutathione showed antioxidant effects by significantly reducing mast cells present in the mucosa of cachectic animals, possibly by attenuating local oxidative stress. This is because the inflammatory process and the production of reactive oxygen species are related to the modulation of mast cell function (Wolfreys and Oliveira 1997) as well as to the activation and degranulation of these cells (Santos et al. 2000; Chelombitko et al. 2016). Furthermore, it has already been demonstrated that the administration of l-glutathione increases the levels of the anti-inflammatory cytokine IL-10 in animals with Walker-256 tumor (Oliveira et al. 2023), thereby strengthening the immune defense and reducing the recruitment of immune cells.

In healthy animals, l-glutathione administration showed a possible pro-oxidant activity, as the results were similar to and/or worse than those of the diseased group. In contrast, l-glutathione acted in reducing cells in apoptosis in response to the reduction of crypt size. And also the decrease of 5-HT + Caspase-3-IR cells in the crypt and lamina propria region shows the preservation of 5-HT-IR cells, related to immune defense, since serotonin promotes the recruitment of innate immune cells (Shajib et al. 2017).

In conclusion, treatment with 1% l-glutathione in cachectic animals demonstrated partial recovery of the damage detected in the intestinal mucosa. More promising results would be observed if the experimental period was extended, which was not possible due to the aggressiveness of the tumor. In healthy animals, the administration of l-glutathione has been controversial, as some results found demonstrated toxicity while others have been beneficial.

## Data Availability

No datasets were generated or analysed during the current study.
